# Using Complementary Acoustic and Optical Techniques for Quantitative Monitoring of Biomolecular Adsorption at Interfaces

**DOI:** 10.3390/bios2040341

**Published:** 2012-09-26

**Authors:** Rupert Konradi, Marcus Textor, Erik Reimhult

**Affiliations:** 1 BASF SE, Advanced Materials and Systems Research, D-67056, Ludwigshafen, Germany; E-Mail: rupert.konradi@basf.com; 2 Laboratory for Surface Science and Technology, Department of Materials, Swiss Federal Institute of Technology (ETH) Zurich, Wolfgang-Pauli-Strasse 10, CH-8093 Zurich, Switzerland; E-Mail: marcus.textor@mat.ethz.ch; 3 Laboratory for Biologically inspired materials, Department of Nanobiotechnology, University of Natural Resources and Life Sciences Vienna, A-1190 Vienna, Austria

**Keywords:** quartz crystal microbalance with dissipation monitoring (QCM-D), surface plasmon resonance (SPR), optical waveguide lightmode spectroscopy (OWLS), dual polarization interferometry (DPI), hydration, polymer brush, supported lipid bilayer (SLB), protein adsorption kinetics, conformational changes, PMOXA

## Abstract

The great wealth of different surface sensitive techniques used in biosensing, most of which claim to measure adsorbed mass, can at first glance look unnecessary. However, with each technique relying on a different transducer principle there is something to be gained from a comparison. In this tutorial review, different optical and acoustic evanescent techniques are used to illustrate how an understanding of the transducer principle of each technique can be exploited for further interpretation of hydrated and extended polymer and biological films. Some of the most commonly used surface sensitive biosensor techniques (quartz crystal microbalance, optical waveguide spectroscopy and surface plasmon resonance) are briefly described and five case studies are presented to illustrate how different biosensing techniques can and often should be combined. The case studies deal with representative examples of adsorption of protein films, polymer brushes and lipid membranes, and describe e.g., how to deal with strongly *vs*. weakly hydrated films, large conformational changes and ordered layers of biomolecules. The presented systems and methods are compared to other representative examples from the increasing literature on the subject.

## 1. Introduction

Biointerfaces—interfaces between synthetic materials and biological systems—constitute one of the most dynamic and expanding interdisciplinary fields in bio-oriented science and technology. Reasons for this include: an increased awareness of the importance of interfacial processes in industrial and clinical applications of materials and devices, ranging from medical implants and tissue engineering scaffolds to *in vitro* and *in vivo* biosensors; the great progress that has been made in the last 10–15 years in specifically tailoring biologically relevant interface properties at the macro, micro and nanoscale through surface functionalization; and the rapidly expanding toolbox of available characterization and sensing techniques. The impact of the latter tools has been two-fold: first, analytical surface characterization techniques, including vacuum-based *ex situ* methods, such as XPS, ToF-SIMS and MALDI, can provide detailed insight into the chemical, structural and morphological properties of surfaces, a prerequisite for the ability to understand how surface properties affect biointerfacial processes. Second, *in situ* techniques provide an important opportunity to monitor the kinetics and pathways of dynamic processes at interfaces under biologically relevant conditions, with the latter ranging from model solutions of simple composition, e.g., single protein solutions, to complex media such as body fluid or cell culture media. 

To understand and master dynamic surface modification and surface recognition processes it is necessary to follow *in situ *interfacial interactions *quantitatively* and in *real time*. In particular self-assembly processes from (aqueous) solution, e.g., self-assembly of functional polymers and lipid-based biomimetic systems, require quantitative monitoring of adsorption/desorption processes in solution, which often include transitions between different macromolecular and supramolecular states. Similar requirements apply to sensing specific and non-specific biomolecular interactions at interfaces in application fields such as genomics, proteomics and glycomics.

Many different *in situ* sensing techniques have been developed in the past, some are regularly used in the biointerface and biointeraction communities, all with their specific pros and cons regarding e.g., ease of use, type of information gained, degree of transducer complexity, detection limits and how quantitative the obtained data is. Sensing techniques that allow the detection of targets without the need for introducing labels are of particular interest, both with respect to costs and in view of potential undesirable changes to the target function induced by the label. Another highly desirable quality of these techniques is the ability to monitor consecutively, *in situ* and in real time, the preparation of a substrate, the surface functionalization steps and the subsequent interaction with biological media; thus ensuring a maximum of reliability and quantitativeness in the correlation of surface properties with biointeraction readout.

The focus of this tutorial review is on the combination of label-free, *in situ*, evanescent-field-based, optical sensing techniques with a quartz crystal microbalance with dissipation monitoring (QCM-D). These techniques will be described and the importance of using two or more such complementary techniques to achieve accuracy in data interpretation will be emphasized. The review covers the measurement principles of the selected methods, the type of information that can be gained by them, their limitations as standalone techniques, and the evaluation and modeling that can be performed to get the most out of typical combined data sets. Examples of specific applications are presented as tutorials with the added value of using a combination of the presented biosensor techniques. We show that such combination measurements do not only serve as a useful cross-check, they also increase quantitativeness and create synergies regarding the type and quality of the information that can be gained, for example allowing new physical properties of a film to be probed and mapping of conformational states. 

Water is often neglected in studies of biomolecular adsorption despite being perhaps the most important “biomaterial”. Hydration of both polymeric surfaces and biomolecular entities in solution and at interfaces is an essential part of their functionality, with coupled water as a crucial factor that affects the interaction of biomolecular adsorption and recognition. We show in *Case Study 1* how we can determine quantitatively the degree of water coupled to self-assembled hydrophilic polymer layers by combining optical waveguide lightmode spectroscopy (OWLS) and quartz crystal microbalance with dissipation monitoring (QCM-D) measurements and how this translates into conformational properties of the polymer and degree of interactiveness with proteins in solution. *Case Study 2* covers a combined surface plasmon resonance (SPR)/QCM-D study of the kinetics of liposome adsorption and spontaneous formation of a supported planar lipid bilayer (SLB) as an example of a biomolecular system undergoing structural transformations. It is demonstrated how QCM-D frequency and dissipation curves with their characteristic signatures for liposome adsorption and SLB formation can help to improve the quantitative interpretation of evanescent optical field techniques and also result in qualitatively new information in terms of the time evolution of the actual surface coverage of the two states. *Case Study 3* discusses the real time modeling of biofilm properties using the example of SLB formation followed by protein binding on top of a biotinylated SLB, the latter being a comparatively simple system representative of the most frequently performed biomolecular adsorption studies. *Case Study 4* highlights the danger of interpreting the kinetics based on the results of a single investigative technique taking the interaction of E. coli total lipid extract liposomes with titanium oxide surfaces as an example. QCM-D and OWLS studies result in apparently contradictory conclusions that could, however, be resolved based on a model of non-planar SPB conformation that takes the different transducer principles into account. Finally, *Case Study 5* demonstrates the potential, rarely exploited so far, of a highly sensitive waveguide spectroscopy using two polarizations, such as Dual Polarization Interferometry (DPI), to probe conformational changes in thin biomolecular films based on their inherent optical anisotropy, by using additional data obtained by complementary techniques.

We close the article with some remarks regarding the implications of the case studies for sensitivity and single-technique kinetics measurements and summarize the trends in the field.

## 2. Surface Plasmon Resonance (SPR)

Surface Plasmon Resonance (SPR) or Surface Plasmon Spectroscopy (SPS) for biosensing was first suggested and demonstrated in 1983 by Lundström and coworkers [1], although it had already been used for several years to study organic layers on metal surfaces [2,3]. In the beginning of the 1990’s SPR started to become wide-spread with the arrival of commercially available, easy-to-use, setups [4,5]. Today, it is one of the standard instruments used in biotechnology research labs and the pharmaceuticals industry and it is the most commonly used technique to study kinetics of biomolecule binding, adsorption and desorption to date [6,7].

In addition to the high sensitivity and temporal resolution offered by SPR, the main advantage over most competing methods, such as fluorescence-based, particle-labeled (e.g., quantum dots) and radioactivity-based enzyme-linked immunosorbent (ELISA) and other assays, is that there is no need to label the target molecules. Thus, a time consuming, costly and perhaps also result-altering step in binding studies is avoided.

**Figure 1:**
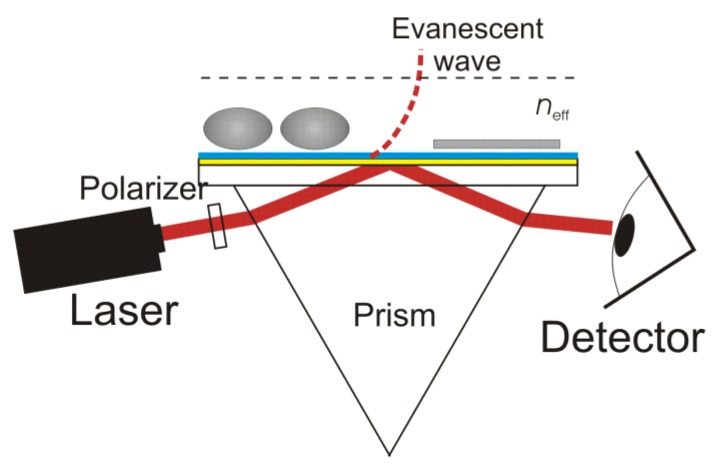
Schematic of a surface plasmon resonance (SPR) measurement in the Kretschmann configuration. The laser beam, totally internally reflected from the back-side of the sensor surface, creates a plasmon coupled to an evanescent field in the solution.

SPR can sense changes in the effective refractive index smaller than 10^−5^ with a time resolution of a few seconds [8]. At lower resolution for changes in the refractive index, the time step is only limited by the temporal resolution of the intensity detector. In the Kretschmann configuration (see Figure 1) [9], the laser excites an electron density wave, called a plasmon, at the surface of a noble metal film (typically gold) through total internal reflection from the back of the sensor surface. The exponentially decaying evanescent electromagnetic field extending from the plasmon into solution concentrates the sensitivity to a volume within ~70–150 nm from the surface [10]. For the plasmon to be excited the momentum of the incoming photons must match the momentum of the surface plasmon. Matching occurs only for *p*-polarized light when [10]

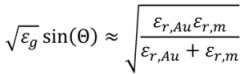
(1)
where *ε*_g_ is the dielectric constant of the prism or glass slide on which the gold film with dielectric constant *ε_r,Au_* is evaporated, and *ε_r,m_* is the dielectric constant of the medium on the other side of the gold film. For this to occur, it follows that *ε*_g_ must be higher than *ε_r,m_*. When Equation (1) is fulfilled, the energy transfer to the plasmon creates a minimum in the reflected intensity of the reflected laser beam. The angle at which this condition occurs depends on the refractive index of the medium for the evanescent wave. Thus, it is possible to sense changes in the medium within the evanescent field caused by adsorption of biomolecules, which changes *ε_r,m_*, by monitoring the change in angle of the incoming light at which the resonance occurs for monochromatic laser light.

In practice, the minimum in reflected intensity corresponding to the plasmon resonance in the Kretschmann configuration can be monitored directly by, e.g., a diode array [11] or by first measuring the entire angle spectrum and then monitoring changes in reflected intensity at a fixed angle or movable detector [12]. 

Interpretation of the SPR response is often done by multiplication of the angle shift with a proportionality constant unique for the adsorbed species and calibrated independently by, e.g., radiolabeling. This is possible for thin films of molecules that do not absorb light at the laser wavelength, or in homogenous distributions of the adsorbed molecules throughout the sensing depth of the SPR. Often, instead of making the sometimes complicated conversion to adsorbed mass, the optical thickness of the adsorbed film is used as a measure of the adsorption. The optical thickness can be described as the film thickness that for a given refractive index of the adsorbed molecules would yield the measured shift in plasmon angle. It is for example typically assumed that *n* ≈ 1.45 is a good approximation for most proteins, although there are clear indications in the experimental and theoretical literature that the refractive index might vary between proteins, due to both their packing and conformation. When an appropriate calibration factor to determine the mass from the SPR angle shift is not known—as mostly is the case when investigating new biointerface interactions—the most appropriate method is to solve for the thickness of the adsorbed film in a multilayer model from the complete intensity *versus* incident angle spectrum using Maxwell’s equations [13,14,15]. This approach takes into account the strong distance dependence, orthogonal to the sensor surface of the intensity of the light. If the assumptions are reasonable, a good approximation of the adsorbed mass can then also be obtained using the de Feijter formula [16], which is described below for optical waveguide lightmode spectroscopy.

As a finat note, it can be mentioned that while commercial SPR setups today are available for high-throughput screening of large arrays, simultaneously using advanced optics and fluidics, recent developments in the research laboratory environment has seen SPR merge with fluorescence spectroscopy and microscopy to provide unprecedented sensitivity as well as distance resolution orthogonal to the surface [17,18,19,20]. In combination with microscopy a high lateral resolution can be achieved only limited by the propagation length of the plasmon (~1-2 μm), however, these advances come at the expense of labeling of the sample and will not be further discussed here.

## 3. Optical Waveguide Lightmode Spectroscopy (OWLS)

**Figure 2:**
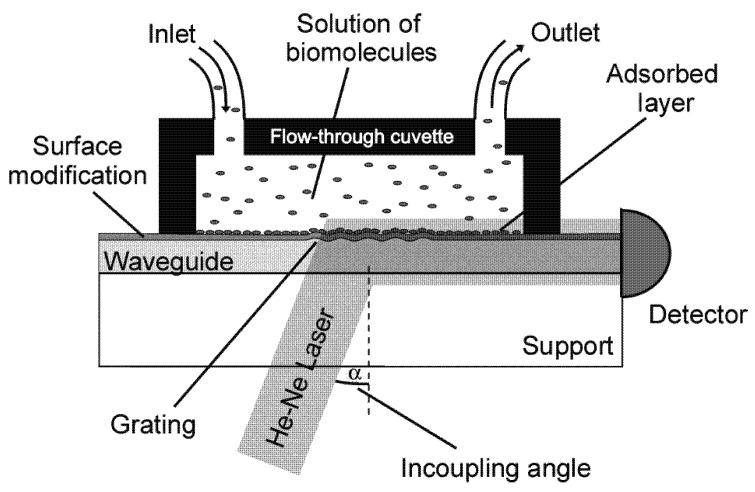
Schematic diagram of the optical waveguide lightmode spectroscopy (OWLS) setup. Light from a He-Ne laser is coupled into the waveguiding film via an optical grating. Only at a specific angle of incidence α, under constructive interference conditions, is a guided mode excited and a peak in light intensity detected. The evanescent field penetrates about 100–200 nm into the bulk solution and senses changes of the refractive index in this region. Thus, molecular adsorption can be precisely monitored by measuring the incoupling angle as a function of time.

OWLS, similar to SPR, is an optical *in situ *technique capable of monitoring, in real time, changes in the polarizability density, *i.e*., in the refractive index, in the vicinity of the waveguide surface [21]. As in SPR, the sensing principle of OWLS is based on an evanescent light field, thus confining the probe depth to a region extending less than a few hundred nanometers above the waveguide. In contrast to SPR, the evanescent field, however, does not originate from a surface plasmon, but from light propagating in a planar optical waveguide (see Figure 2).

An optical grating, typically with a modulation of ~10 nm at a periodicity of ≤500 nm etched or embossed into the waveguide surface [22], serves to couple the monochromatic laser beam into the waveguide. Commercially available waveguide sensors have been made by vacuum-based deposition or sol-gel processes, producing highly transparent waveguiding layers composed, for instance, of silicon oxide (SiO_2_), titanium oxide (TiO_2_), tantalum oxide (Ta_2_O_5_) or mixed SiO_2_/TiO_2_ [23]. The waveguiding layer can be further surface-modified e.g., by reactive magnetron sputter deposition of a nanometer-thin layer, thus allowing for the study of (bio)molecular interaction with specific substrate chemistries; for example, thin coatings of Nb_2_O_5_ or TiO_2_ have been successfully used as model surfaces that resemble biomedically relevant metal implant surfaces [21]. 

To excite a guided mode, the phase shift upon total internal reflection needs to equal zero. This condition, for mono-mode waveguides, is only fulfilled at two distinct angles for the two polarization modes, transverse electric and transverse magnetic. Since the evanescent field is sensitive to changes in the refractive index close to the waveguide surface, the condition for constructive interference changes upon (bio)molecular adsorption; thus, by precisely measuring the coupling angle as a function of time, such adsorption/desorption processes can be studied in real-time and quantitatively. Pre-coating of the waveguides similarly affects the condition for constructive interference and one has to ensure that the corresponding coupling angles are still within the instrumentally accessible range. In particular, when the refractive index of the coating material is higher than that of the waveguide itself, as is the case for TiO_2_ and Nb_2_O_5_, it has been shown that the overcoat has to be below about 10 nm [21]. Note that the sensitivity is limited to the area of the grating on the waveguide and that a coating on the surface can change the penetration depth of the evanescent field.

An advantage of OWLS over SPR is that, by monitoring both polarization modes, two parameters, the thickness and the refractive index of the adsorbed layer, can be simultaneously determined. Thus, no assumption, e.g., of the refractive index, has to be made and the surface adsorbed mass density *m_adsorbate_* (typically given in ng/cm^2^) can be directly calculated according to de Feijter’s formula [16]

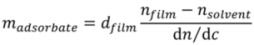
(2)
together with the adlayer thickness and refractive index, *d_film_* and *n_film_*, respectively, the refractive index of the bulk medium *n_solvent_* and the refractive index increment d*n*/d*c*. Using a benchtop refractometer, d*n*/d*c* can readily be measured for any specific combination of analyte and buffer. For most protein adsorption studies it is a fair approximation to use a d*n*/d*c* value of 0.18 cm^3^/g [24]. 

In summary, similar to SPR, OWLS allows for an on-line monitoring of (bio)molecular adsorption and desorption processes in real-time without the need for any labeling procedure. OWLS is highly sensitive with a detection limit <1 ng/cm^2^ and a time resolution of a few seconds [25]. Due to the simultaneous determination of the adlayer film thickness and refractive index, the correct determination of *m_adsorbate_* is even possible when (de)hydration, denaturation or other processes lead to changes in the density and concomitantly the refractive index of the adlayer during the adsorption process. It has to be emphasized though that in the region of films thinner than a fraction of the wavelength of light as typically studied by OWLS, the thickness and the refractive index cannot be well separated, however, well their product is determined. This is similar to ellipsometry, for which the same restrictive thin film limit applies [26]. Like SPR, OWLS is a mean-field technique that generally averages information from several mm^2^ determined by the beam and grating size. With special optics, lateral resolutions of about 100 µm can be achieved. Furthermore, OWLS, like SPR, relies on the assumption of a homogenous surface coverage and an isotropic refractive index. Recent developments in OWLS have been mostly directed towards increasing the sensitivity by either exploiting changes in the intensity of the outcoupled light when using light absorbing adsorbates (e.g., fluorescently labeled proteins) [27,28] or by exploiting the surface-confined fluorescence excitation by the grating coupler evanescent field [29,30,31]. Both approaches necessitate labeling but allow for decreasing the detection limit by 1–2 orders of magnitude in comparison to standard OWLS and conventional fluorescence microscopy. Similarly, optical waveguides have been used for the total internal reflection fluorescence (TIRF) excitation of microarrayed surface-bound fluorophores [32]. Another recent improvement of standard OWLS regards the probe depth. It has recently been extended to layer thicknesses of up to 400 nm by using an extended model for the mode equations [33]. It has also recently been combined with phase sensitive readout to reach a sensitivity of 1 pg/mm^2^ and applied to studying molecular orientation in thin films [34] similar to what will be described in Case Study 5 using another waveguide technique below. It has also been combined with electrochemical sensing for simultaneous control and measurement of electrochemical processes at the interface using transparent ITO electrodes [35,36].

## 4. Quartz Crystal Microbalance (QCM)

**Figure 3:**
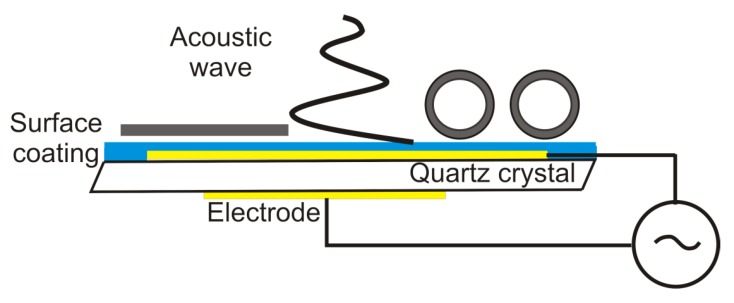
Schematics of the quartz crystal microbalance. An alternating electric field applied across a piezoelectric quartz crystal yields a resonant shear acoustic wave in the sensor crystal. The shear oscillation couples to the adsorbed film on top and shifts the resonant frequency and energy dissipation in response to the added mass and viscoelastic load.

The QCM sensor is made of a thin piece of quartz, which is a piezo-electric material. An oscillating electric field is applied across the crystal typically making it deform in thickness-shear mode, *i.e.*, perpendicular to the applied electric field (Figure 3). Each crystal has a fundamental resonant frequency determined by 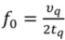, where *ν_q_* is the speed of shear waves in the crystal and *t*_q_ is the thickness of the crystal. When the driving electric field oscillates at the fundamental frequency the mechanical amplitude of the crystal shear oscillation is increased more than hundred-fold. The width of the crystal resonance is very narrow, which gives an extremely well-defined resonant frequency and the ability to measure changes in the resonant frequency very precisely. Since the resonant frequency is determined by the total oscillating mass, which also includes all mass that is coupled to the surface, it can be used to measure the mass adsorbed on the surface in real time without need for any labels. A good approximation for the mass adsorbed to the sensor surfaces is the Sauerbrey relation [37]:

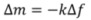
(3)
where *k* is the mass sensitivity of the crystal (~18 ng/(Hz × cm^2^ for a typically used 4.95 MHz crystal). Resonance is also observed at so-called overtones of the fundamental resonant frequency, which respond to changes in mass in a manner similar to the fundamental frequency. Only odd-numbered overtones can be produced, yielding overtone resonances at, e.g., 15, 25, 35, … MHz for a 5 MHz crystal. The conditions that have to be fulfilled for the Sauerbrey relation to be strictly valid are that the mass of the adsorbed film must be: (i) much smaller than the mass of the crystal (<5%); (ii) evenly distributed over the active area of the sensor or with an inhomogeneous but symmetric and periodic distribution around the geometrical center of oscillation; (iii) rigidly attached to the surface (no slip); (iv) complete bulk rigidity (no visco-elastic deformation induced by the sensor motion in the overlayer to be measured); Of these, criterion (v) can mostly not be relaxed for biological molecules or polymers adsorbed from aqueous solution, while the other assumptions are less critical. 

An acoustic sensor operated in liquid will lose the mechanical energy stored in the oscillation at a much higher rate than when operated in gas or vacuum, since molecules in the liquid environment move with the oscillating surface. The magnitude of these losses depends on the density and viscosity of the liquid. The extinction depth of the shear wave is given by 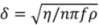, where *η* is the viscosity and *ρ* the density of the medium, *i.e*., typically water, and *f* the fundamental resonance frequency of the crystal [38]. For a 4.95 MHz crystal in water this yields *δ* ~ 250 nm for the fundamental resonance frequency, and as can be seen the extinction depth will decrease approximately as the inverse square root of the overtone number, *n*. Important to note is that the decay length is strongly dependent on the medium and an adsorbed film can significantly increase the decay length and therefore the probing depth of the acoustic sensor.

An important property of acoustic sensors in general is that they measure all mass coupled to the oscillation and this mass to a large part in a liquid environment will include liquid trapped in or exchanged with the adsorbed film. Thus, not only dissipation within the viscous molecules, but significantly, also losses from the viscous liquid flowing in the film will affect the crystal oscillation. The high dissipation of energy in these systems implies that the validity of the Sauerbrey relation should frequently be questioned when a QCM is operated in liquid. However, the changes in energy dissipation, Δ*D*, can be measured in real time either from the width of the resonance peak in the frequency domain [39] or from the decay constant of the shear amplitude of the freely oscillating crystal in the time domain [40]. Operating the QCM in the latter fashion to obtain the energy dissipation is referred to as the quartz crystal microbalance with dissipation monitoring technique (QCM-D) [40,41,42]. By simultaneous measurements of resonance frequency and energy dissipation changes, the internal structure of the film can be probed by applying a visco-elastic model [39,43,44]. Quantitatively, the commonly quoted criterion for when models accounting for losses should be applied instead of the Sauerbrey relation is: 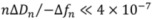 Hz^−1^ for a typical 4.95 MHz sensor crystal. 

Typically, Voigt-Kelvin elements are used to represent the adlayers and by simultaneous measurements at several different overtones, film mass, elasticity and viscosity can be calculated [43,44]. The Voigt-Kelvin model assumes a frequency independent complex shear modulus, which should seldom be expected to be the case at the operation frequencies of a QCM, or for that matter from biopolymer films tethered to a sensor surface [45]. Despite this it is the currently most used model and fulfills its main purpose of correcting adsorbed mass estimates from viscous losses. Recently it was shown that the origin of losses in films with tethered molecular complexes can be attributed to hydrodynamics, and give rise to a much more complex loss behavior than expected from uniform visco-elastic overlayers as in the commonly applied Voigt-Kelvin model [46]. The requirement to perform hydrodynamic (finite element) modeling to catch such effects still makes visco-elastic modeling attractive as a first-order approach to correct for visco-elastic losses in the determination of adsorbed mass using e.g., the Voigt-Kelvin model. An excellent summary of the interpretation of QCM data when viscous losses are present is given by Reviakine *et al*. in [45].

## 5. Comparing Acoustic and Optical Evanescent Techniques

Evanescent optical methods are often easy to use and give a direct measure of the number of adsorbed molecules with high temporal resolution since they measure the change in optical contrast when molecular adsorbates replace liquid close to the sensor surface. On the flip side, they suffer from a lack of independent conformational information, making it hard to obtain the right mass adsorption kinetics for species undergoing large conformational changes and even harder to clearly map out intermediate states of an adsorption process which do not change the mass adsorption rate to the surface. Furthermore, if the adsorbed film displays molecular alignment, this typically affects the conversions used for calculating film thickness and mass from the measured changes in refractive index due to the common use of linearly polarized evanescent fields, as effective refractive indexes determined in the bulk are based on random orientations of anisotropically polarizable biomolecules. This is usually not taken into account in single-technique measurements, but can lead to large errors that will be addressed later.

**Figure 4:**
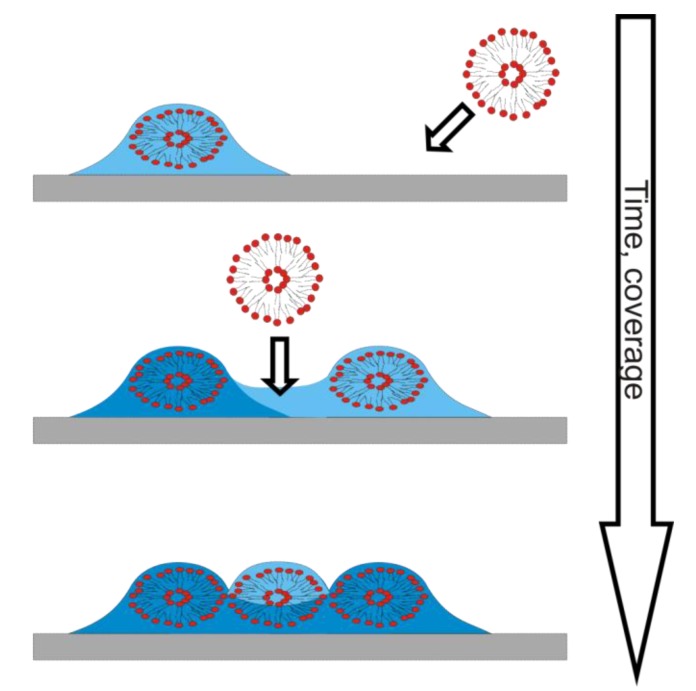
The quartz crystal microbalance with dissipation monitoring (QCM-D) includes water coupled to the adsorbed film in its measured mass. The amount of water coupled per adsorbed molecule varies with surface coverage (time). The dark blue areas symbolize already sensed biomolecule and water mass, while the light blue areas symbolize additional mass sensed after further biomolecule adsorption.

Acoustic techniques, like QCM-D, can with the state-of-the-art instrumentation available today provide a richness of conformational information of adsorbed films. However, they suffer from measuring a response proportional to the total (dynamic) mass load on the sensor. The total mass load is in most cases not proportional to the actual number of adsorbed molecules, due to coverage dependence in the amount of coupled liquid, as schematically depicted in Figure 4. This obscures the true mass adsorption kinetics even for simple adsorption processes, but can, when properly understood and addressed with complementary measurements, lead to greater understanding of the properties of the adsorption process and the adsorbed thin films. Several aspects of this will be demonstrated in the following case studies.

Both acoustic and optical evanescent field sensor responses are interpreted with mean-field theories, which average properties across the sampled surface. Thus, the sampled area is important to either detect or avoid fluctuations and inhomogeneities in the state of the adsorbed films. Generally, a sampled area is chosen which is macroscopically large in order to average out fluctuations. Fluctuations will be present in all systems where interaction-triggered conformational changes occur. In order to correlate the averaged film properties to molecular or supramolecular states in the film, microscopy methods like confocal laser scanning microscopy (CLSM) or, preferably, atomic force microscopy (AFM) are typically employed. These methods, on the other hand, mostly have a low time-resolution, are technically demanding and time-consuming, risk influencing the sample by the interaction with the AFM tip and are limited in probing areas for which statistics are low and fluctuations could become important. The significant differences between microscopic techniques and mean-field biosensor techniques often make direct comparisons difficult.

## 6. An Overview of the Application of Complementary Sensor Techniques

Due to the imposed limitations for single-technique measurements outlined in the previous section, it has been increasingly common to use more than one technique to characterize a sample to exclude artifacts. There are several research groups that have taken a more comprehensive approach where not only more than one technique is used, but where mutually complementary biosensing techniques are used together with models that increase the information content from the measurements beyond that provided by the separate techniques on their own. Despite rapidly gathering momentum, these approaches are not yet as widespread as they should be. Thus, we would like first to briefly review some different systems and approaches that have been attempted by early adopter groups, before using a few examples to illustrate the principles of how biosensors can be combined to study thin films with a high degree of conformational complexity. This is not a comprehensive review of all such work in the field, but instead serves to highlight different approaches with the main emphasis on the first demonstrated examples using the sensor techniques described above.

Friedt *et al*. describe an instrument combining SPR in Kretschmann configuration with a surface acoustic wave (SAW) sensor [47]. Application examples include the determination of the water content and *in situ* thickness of human immunoglobulin G upon adsorption onto a hydrophobically modified surface [48] and the dependence of the water content, density and thickness of physisorbed poly(*N*-isopropylacrylamide) upon variations in temperature. Johannsmann and coworkers developed a combination instrument using an optical grating etched into a quartz crystal and covered with a gold electrode for simultaneous SPR and QCM measurements [49]. This instrument was applied to study, for instance, streptavidin binding to biotinylated surfaces [49], adsorption and desorption of alkanethiol self-assembled monolayers [12], electropolymerization of pyrrole on gold [12], and the physisorption and desorption of poly(*N*-isopropylacrylamide) and its swelling and de-swelling as a function of temperature [50]. 

Kasemo, Höök and coworkers elaborated a Voigt-model based analysis of the viscoelastic thin film properties sensed by QCM with dissipation monitoring (QCM-D) [43,44,51]. Complementary information from SPR, QCM-D and ellipsometry allowed them to study conformational changes in the adsorbate molecules associated with variations in layer density, thickness and water content [14,44,51,52,53]. For instance, they followed the adsorption of the mussel adhesive protein Mefp-1 and the subsequent cross-linking with NaIO_4_ that lead to a densification and dehydration of the proteinaceous adlayer [44]. Following this work they extended the modeling of combined SPR and QCM-D data (including simultaneously acquired data sets) to determine water content and thickness of protein layers, lipid layers and DNA upon hybridization [14,15,52] as well as protein-DNA binding and DNA replication [53]. This approach will be used for the examples treated at length in the following sections. 

Others followed similar lines in combining QCM-D with an optical technique to study protein-surface interactions [54]. Examples include the dependence of fibrinogen adlayer thickness, hydration and orientation on surface roughness [55], the dehydration of surface-adsorbed fibrinogen upon thrombin exposure [56], the dependence of laminin hydration upon surface hydrophobicity [57] and the orientation of surface-adsorbed artificial leucine zipper proteins [58]. The order and rigidity of streptavidin adsorbed on biotinylated surfaces, the thickness and viscoelasticity changes upon biotin-DNA, subsequent c-DNA binding and specific protein binding mode to DNA were all investigated by Su and coworkers [59,60,61]. Richter and Brisson also studied supported lipid bilayers using a combination of AFM, QCM-D and ellipsometry [62]. Furthermore, Stålgren *et al*. studied surfactant adsorption on hydrophilic and hydrophobic surfaces by comparing QCM-D to *in situ *ellipsometry [63]. Growth of thick polymer brushes has been studied visco-elastically and morphologically using QCM-D, ellipsometry and AFM [64]. 

Finally, several research groups applied complementary techniques to study polyelectrolyte layer-by-layer (PEM) deposition. Picart *et al*. used a combination of OWLS, QCM-D and confocal laser scanning microscopy to unravel the exponential-like growth of poly(*L*-lysine)/hyaluronan multilayer films [65,66]. Halthur *et al*. investigated the layer-by-layer deposition of poly(*L*-glutamic acid) and poly(*L*-Lysine). By comparison of *in situ* ellipsometry and DPI with QCM-D, they observed that the thickness increased linearly while the mass increased more indicating that during deposition the multilayer increasingly expelled water and densified [67,68]. Also Moya and coworkers studied the buildup of poly(diallyldimethylammonium chloride)/poly(sodium styrenesulfonate) and poly(allylamine hydrochloride)/poly(sodium styrenesulfonate) in detail by QCM-D and spectroscopic ellipsometry to elucidate the dependence of the PEM layer growth on hydration [69]. As the acoustic and optical combinations are particularly powerful in determining hydration, the swelling of hydrogels under the influence of ions and proteins has been investigated by several others in e.g., [44,70,71].

Recently, there has been an increasing focus on the combination of ellipsometric [26,44,51,62,69,72] and reflectometric [73] techniques with QCM-D, due to the advent of a commercial setup which allows optical access to the QCM sensor surface in a stable measurement environment. This greatly facilitates real-time combination measurements simultaneously on the same substrate and thereby reduces uncertainties stemming from sample preparation [73]. The methodology applied to combining ellipsometry with QCM-D is essentially the same as the previously developed framework for combining evanescent optical sensing techniques with QCM-D that will be illustrated by case studies below. However, ellipsometry has the advantage of being a high resolution technique applicable to most substrates as the probing beam does not have to pass through the substrate or require special structures on the sensor surface for coupling. However, care has to be taken since the typical QCM sensor substrate is semi-transparent with a complex structure of metal films and birefringent quartz that can affect the accuracy of measuring thick overlayers. The ellipsometry combination with QCM-D has recently been notably explored for a range of different systems and theoretically described by Rodenhausen *et al*., e.g., for studying polyelectrolyte brushes and protein adsorption [70], micelle adsorption [74] and self-assembled monolayer formation [75], as well as with reflectometry, extensively for supported lipid bilayers by Svedhem *et al*. [73,76,77,78]. Despite the recent increase in publications combining ellipsometry with QCM-D *in situ*, the great majority of labs are still combining separate setups of evanescent optical sensing techniques and QCM-D as a powerful combination to combine hydrated organic thin films.

## 7. Case Studies on Using Complementary Data Sets Obtained by Evanescent Optical and Acoustic Sensing Techniques

As described above there are many different interfaces of relevance for biosensing where real-time data acquisition by combinations of optical and acoustic evanescent techniques is useful and has also been applied. To highlight a few different kinds of systems as well as combinations of techniques we will use case studies from our own research to describe how the obtained complementary information can be used to its fullest. Our focus is on describing how these approaches can be used to obtain information of the molecular or film structure at the biosensor interface.

### 7.1. Case Study 1: Measuring Layer Thickness and Hydration by Complementary Evanescent Optical and Acoustic Techniques—A Case Study on PLL-g-PMOXA

**Scheme 1:**
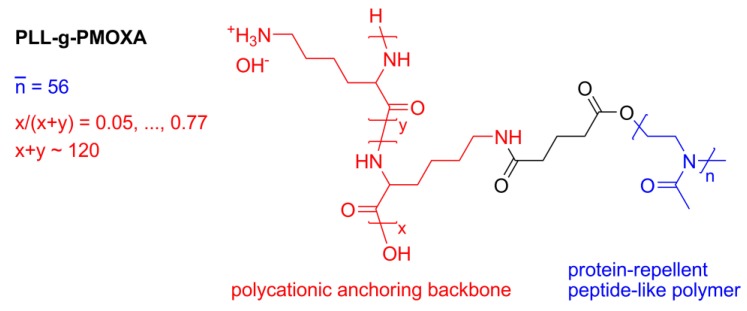
Poly(*L*-lysine)-graft-poly(2-methyl-2-oxazoline).

For the first case study we will investigate ultrathin hydrophilic polymer adlayers with the aim of relating molecular architecture, water content and resistance to protein adsorption. Important properties to characterize towards this aim are the layer thickness and hydration, for which a combination of evanescent optical sensing and acoustic sensing is highly suited. As an example, we employed OWLS and QCM-D to study the surface self-assembly and subsequent exposure to human serum of graft copolymers consisting of a polycationic poly(*L*-lysine) (PLL) backbone and poly(2-methyl-2-oxazoline) (PMOXA) side-chains (PLL-*g*-PMOXA, see Scheme 1).

We have previously shown that PLL-*g*-PMOXA, and analogous copolymers having poly(ethylene glycol) side-chains (PLL-*g*-PEG), self-assemble onto negatively charged surfaces through multiple electrostatic interactions between the positively charged backbone and the surface [79,80,81,82,83]. Depending on the exact grafting ratio *g*, where g is the number of lysine units per side-chain, the hydrophilic grafted chains form a swollen polymer brush and render the surface highly resistant towards protein adsorption [81]. OWLS and QCM-D are employed to determine the adsorbed polymer mass. The data from these complementary techniques is then used to infer through some simple considerations the hydration, the swollen thickness of the brush and the side-chain stretching. Finally we will relate the latter quantity to the adlayers capability to resist non-specific protein adsorption.

**Figure 5:**
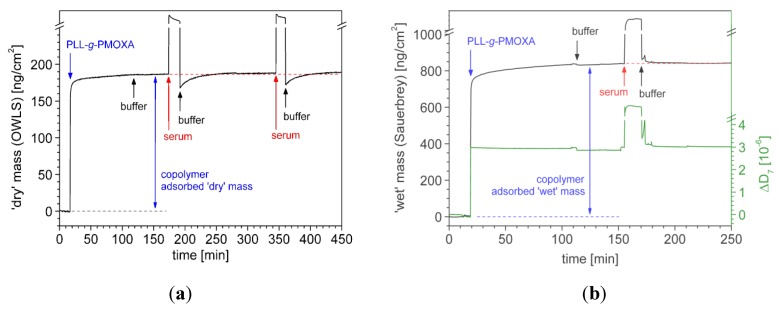
*In situ* investigation of PLL-*g*-PMOXA (α = 0.22) copolymer adsorption and serum exposure by (**a**) optical waveguide lightmode spectroscopy (OWLS) and (**b**) quartz crystal microbalance (QCM-D). Nb_2_O_5_-coated waveguides and quartz crystals were first incubated with the polymer in a physiological buffer solution (10 mM HEPES + 150 mM NaCl adjusted to pH 7.4) and then exposed to full human blood serum for 15 min. The surfaces were rinsed with buffer before and after each adsorption step. In (**b**) the ‘wet’ mass (—) is calculated from the inversely proportional normalized frequency shift by using the Sauerbrey relation, Equation (3). The increase in dissipation (—) upon polymer adsorption is related to an increased viscoelasticity of the adlayer. The kinks in the curves are due to temperature instabilities upon solution injection.

Figure 5 shows typical OWLS (a) and QCM-D (b) experiments for PLL-*g*-PMOXA of medium grafting density. In both cases, first the polymer adsorption was followed *in situ *until a stable layer had formed. Subsequently, after rinsing with buffer, the surfaces were exposed to full human serum (control serum, Precinorm^®^ U, LOT: 171 074-01, reconstituted in ultrapure water) for 15 min and rinsed again with buffer. Comparison of the mass values of the polymer coating equilibrated with buffer solution before and after serum exposure allows the researcher to quantify the adsorbed serum mass.

Since the optical technique OWLS only senses differences in refractive indexes, any water associated with the polymer does not contribute to the calculated mass. Essentially, OWLS measures the molecular or ‘dry’ mass *m_OWLS_* of the adsorbate. In contrast, QCM-D is based on acoustic principles and, hence, is sensitive to the viscoelasticity of the adsorbate. While the shift in resonance frequency Δ*f* for a rigid adlayer is proportional to the adsorbed mass, for soft, viscoelastic films, the energy dissipation in the film has to be taken into account. The steep increase in the dissipation Δ*D* upon PLL-*g*-PMOXA adsorption indicates that the polymer adlayer indeed is highly viscoelastic, presumably due to the strong swelling of the flexible hydrophilic PMOXA side-chains in the aqueous buffer. We analyze our data according to the Voinova model, which assumes a uniform viscoelastic adsorbed film represented by a Voigt-Kelvin viscoelastic solid in contact with a semi-infinite bulk Newtonian liquid under no-slip conditions [43]. Importantly, the acoustic mass thus determined, *m_Voigt_,* includes the water coupled to the film by direct hydration and viscous drag and, hence, effectively measures what is often referred to as the ‘wet’ mass of the adsorbate [44]. 

**Figure 6:**
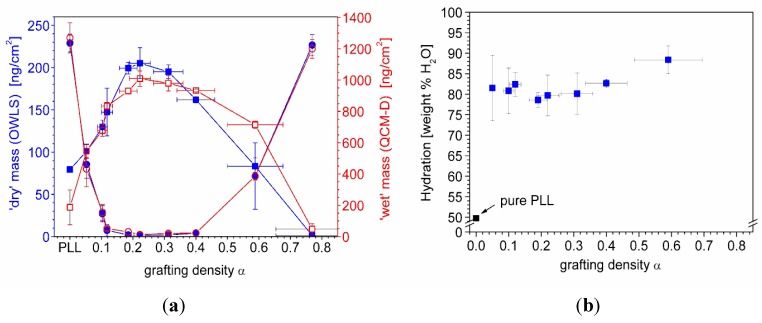
(**a**) Comparison of ‘dry’ mass of adsorbed copolymer (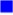) and serum proteins (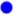) as obtained from OWLS measurements (see Figure 5(a)) and ‘wet’ mass of adsorbed copolymer (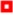) and serum proteins (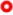) as obtained from QCM-D measurements using Voigt modelling (see Figure 5(b)) for PLL-*g*-PMOXA graft copolymers of varying graft density. α = 0 corresponds to pure PLL. Copolymer and serum adsorbed masses were taken after rinsing with buffer after a stable value was reached; (**b**) Hydration in weight percent of water in the PLL-*g*-PMOXA adlayers calculated from the data in (a) according to Equation (4).

Similar experiments were conducted for a set of PLL-*g*-PMOXA of varying grafting density α, where α denotes the number of PMOXA side-chains per lysine unit of the graft copolymer backbone (α = 1/g). In Figure 6(a), the values obtained for the dry polymer and serum adsorbed masses are plotted together with the corresponding wet masses as a function of the grafting density. The curves show a typical behavior: At low grafting densities, increasing the number of side-chains naturally increases the adsorbed copolymer mass, whereas, at high grafting densities, thermodynamic and kinetic reasons such as the diminished electrostatic attraction and a strong side-chain crowding effectively oppose copolymer adsorption [81,84].

In Figure 6(b) we plot the hydration

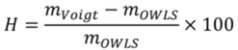
(4)
as a function of the grafting density. Interestingly, all coatings are hydrated to approximately 80% or higher, reflecting the similar shape of the dry and wet mass *versus* grafting density curves in (a). Both, below and above the optimal grafting density, the hydration increases slightly, indicating a marginally lower polymer volume fraction. Strong hydration has been suggested to be a prerequisite for obtaining highly protein-repellent coatings [85,86]. Here, we can certainly corroborate this concept. However, it is also obvious that hydration alone is not a sufficient criterion. Both at low and high grafting densities stable polymer coatings were found that showed a similar or even higher hydration but nevertheless were not resistant to protein adsorption. 

To relate resistance to protein adsorption to the surface conformation and spatial packing constraints of PLL-*g*-PMOXA coatings of different grafting density we therefore estimated the degree of side-chain stretching using the OWLS and QCM-D data. Assuming a two-dimensional hexagonal (close-packed) arrangement of the side-chains on the surface, the mean distance between two chains *L* can be readily calculated from the average side-chain surface density *σ_PMOXA_* according to [79]:

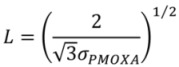
(5)
*σ_PMOXA_* is determined by the adsorbed dry mass *m_OWLS_*, the grafting ratio *g*, the molecular weight of a lysine monomer unit *M_Lysine_* = 128 g/mol and the average molecular weight of the side-chain polymer *M_PMOXA_* = 5,000 g/mol according to [81]

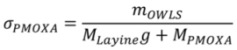
(6)

The volume of the film probed by the QCM is by definition

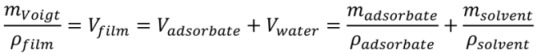
(7)

Equation (7) can be rearranged as

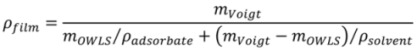
(8)
where *m_OWLS_* is substituted for *m_adsorbate_* and the mass of the buffer trapped inside the layer *m_solvent_* can be obtained from the difference in adsorbed mass measured by QCM-D, *m_Voigt_*, and adsorbed mass measured by OWLS, *m_OWLS_*, and thus substituted for *m_solvent_* [14,51,52]. 

The density of the PLL-*g*-PMOXA part of the adlayer was approximated using the literature value *ρ_adsorbate_* = 1.14 for poly(2-ethyl-2-oxazoline) (Values published online by Scientific Polymer Products, Inc. The density of poly(2-methyl-2-oxazoline) has not yet been reported in the literature). Note that even an unreasonably high error of 50% in *ρ_adsorbate_* affects the side-chain stretching by less than 10%. Then *ρ_solvent_* is close to one for a physiological buffer solution and having *ρ_film_* and *m_Voigt_* one can readily obtain an estimate for the swollen layer thickness [44]

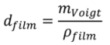
(9)

Finally, the ratio

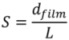
(10)
gives a direct indication for the degree of stretching of surface-bound PMOXA side-chains.

**Figure 7:**
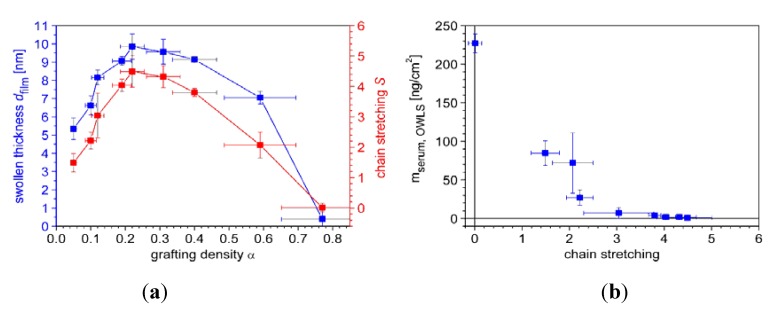
(**a**) Swollen thickness and stretching of PMOXA side-chains as a function of the grafting density calculated using *m_OWLS_* and *m_Voigt_* of the PLL-*g*-PMOXA coatings (see text); (**b**) The ‘dry’ serum adsorbed mass decreases as a function of the side-chain stretching. Full resistance to protein adsorption is reached for chain stretching of approximately S ≥ 4.

In Figure 7(a) *d_film_* and *S* are plotted as a function of the PMOXA side-chain grafting density. At low grafting densities, up to a value of α ≈ 0.22, both quantities increase approximately linearly with grafting density up to maximum values of *d_film _*≈ 10 nm and *S* = 4.5. In this regime also the adsorbed polymer mass shows a linear increase with grafting density (see Figure 6(a)) and is mainly determined by the number of PMOXA side-chains per PLL backbone. This increase in side-chain density at more or less constant backbone density causes the corresponding increase in chain stretching and swollen thickness. At high grafting densities where the polymer adsorption becomes limited, decreasing swollen thickness and decreasing chain stretching are observed with increasing grafting density. Since OWLS and QCM are mean-field techniques, the measurements cannot be laterally resolved and the quantities *m_OWLS_* and *m_Voigt_* are average values over the entire surface area. In Equations (5) and (6) the surface number *σ_PMOXA_* and spacing *L* of PMOXA side-chains were calculated based on *m_OWLS_*, inherently assuming a homogenous distribution of PMOXA side-chains. Thus, any molecular inhomogeneity of the polymer surface coverage, side-chain spacing and stretching are not accounted for in these calculations. Therefore, in the high grafting density regime, where stiffening of the backbone may cause lateral inhomogeneities, the calculated swollen thickness and side-chain stretching would be averaged over densely grafted adsorbed copolymer molecules and uncovered surface area. Thus, these values might not reflect the swollen thickness and chain stretching on a molecular scale.

Finally, in Figure 7(b) the serum adsorbed mass as determined by OWLS is related to the side-chain stretching. High resistance to protein adsorption is obtained for chain stretching of approximately *S* ≥ 4. In summary, by utilizing the difference in transducer principles of OWLS and QCM-D for monitoring polymer brush and protein adsorption we could calculate not only the water content of the polymer brush, but also the chain stretching and show that it is the latter property which correlates with suppression of protein adsorption.

### 7.2. Case Study 2: How to Reveal Adsorption Kinetics of Biomolecular Systems Undergoing Structural Transformation—A Case Study on SLB Formation

In addition to determining the mass, hydration and conformation of, e.g., a polymer layer, a combination of evanescent optical and acoustic sensing techniques can be used to probe conformational states of a film in real time. An adsorption process under intense study for a decade has been that of small unilamellar liposome adsorption and subsequent spontaneous rupture and formation of a supported planar lipid bilayer (SLB) [15,87,88,89,90,91,92]. The liposome/SLB system undergoes a large conformational change, transforming a bulky, water containing, viscous, supramolecular, complex with a wide distribution of molecular orientations (liposomes) into a rigid, highly oriented structure (SLB), with the two phases coexisting on the surface during a major part of the adsorption process. However, it is known that up until a certain surface coverage of liposomes no rupture to form SLB will take place for the process of e.g., palmitoyl-oleoyl-phospocholine (POPC), dioleoyl-phospocholine (DOPC), or mixed POPC or DOPC and plamitoylphosphoserine (POPS) or dioleoylphosphatidylserine (DOPS) liposomes adsorbing to SiO_2_ (the latter only in the presence of divalent cations). It was impossible for a long time to obtain the correct mass uptake or surface coverage kinetics with a single technique and the first such determination was obtained instead by a real time combination of SPR and QCM-D data, which made it possible to separate the response of the two phases (liposomes and planar bilayers) [14,15].

Because of the exponential decay of the sensitivity of the order of 100 nm from the surface of a SPR sensor the distribution or thickness of a layer is important for an accurate determination of adsorbed mass. This problem is mostly avoided by use of waveguide spectroscopy where even if thickness and refractive index cannot always be independently calculated, their product proportional to mass can be determined with quite high accuracy by the simultaneous measurement of the response using two polarizations of the evanescent field. The main exception to this is for optically anisotropic films, which will be discussed in more detail below. This is important to emphasize since a SLB is optically anisotropic and thus it is not straightforward to analyze by waveguide spectroscopy alone.

**Figure 8:**
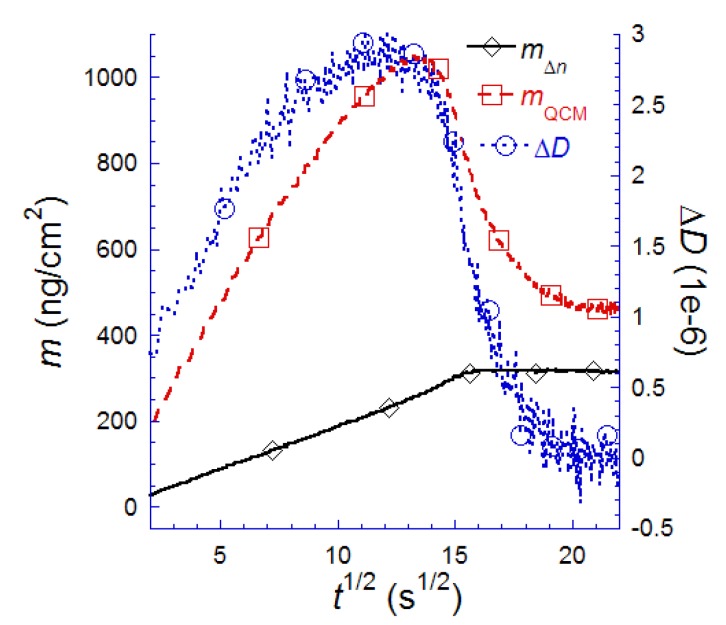
Mass adsorption kinetics measured simultaneously by QCM-D and SPR, respectively, for the POPC SLB formation process on SiO_2_. The masses are calculated using the Sauerbrey relation for *m*_QCM_ and the assumption of a uniformly thick film throughout the adsorption process and a d*n*/d*c* ≈ 0.25 for the lipids in an SLB in a p-polarized field [93]. Adapted with permission from Reimhult *et al*. [14]. *Anal. Chem*. **2004**, *76*, 7211–7220. Copyright 2004 American Chemical Society.

It was described in the SPR and OWLS sections that changes in the thickness of the adsorbed film, *d*_film_, and d*n*/d*c* affects the mass calculation from the SPR response, by the influence of *d*_film_ on the solution to the equations describing the propagation of light through the multilayer structure to determine *n* and the influence of both *d*_film_ and d*n*/d*c* on Equation (2) [14,15]. Both these parameters are very different for vesicles and SLBs, where *d*_vesicle_ ~ 50 nm, *d*_SPB_ ~ 5 nm, and the difference in d*n*/d*c* will vary depending on lipid composition and deformation of adsorbed liposomes and ordering of lipids in the SLB. Thus, we have to calculate the respective masses separately, by first separating the transducer signal, ΔΘ, into one for each species. Inspection of Figure 8 yields that Δ*D* is essentially zero for the SLB formed in the asymptotic limit and it has been demonstrated that the time where the maximum in Δ*D *occurs is a good estimate of the time at which the critical coverage needed for vesicle rupture is reached [94]. Since only liposomes contribute to changes in Δ*D* and since the surface is covered only by vesicles up to the critical coverage, Δ*D* will be used to separate the lipid molecule mass-response of vesicles and SLB in the SPR signal, ΔΘ. 

**Figure 9:**
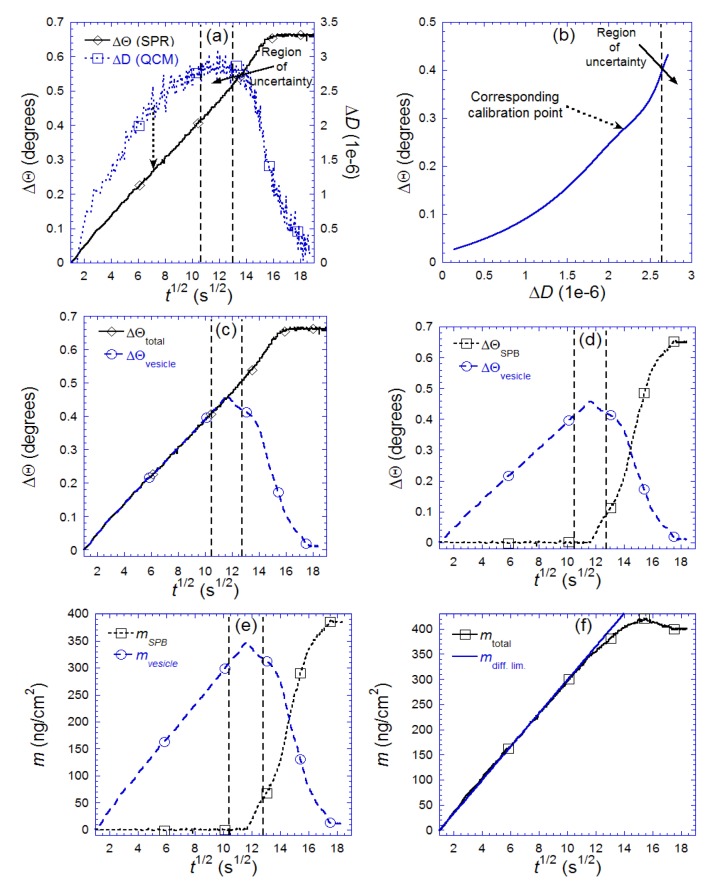
Illustration of the step-by-step procedure (**a–f** described in the text) used to separate the contribution to the change in SPR angle, Θ, and coupled mass, *m*_total_, originating from adsorbed vesicles (ΔΘ_vesicle_, *m*_vesicle_) and supported bilayer islands (ΔΘ_SPB_, m_SPB_), respectively. Also shown in (**f**) is the expected mass adsorption from diffusion limited transport of lipid material in liposomes. Adapted with permission from Reimhult *et al*. [14]. *Anal. Chem*. **2004**, *76*, 7211–7220. Copyright 2004 American Chemical Society.

Figure 9 details a step-wise procedure allowing the separation of ΔΘ_total_(*t*) into its two components: ΔΘ_vesicle_(*t*) and ΔΘ_SPB_(*t*) (Figure 8(a–d)). Since the surface is occupied only by vesicles until the critical coverage is reached [15,94], the temporal variations in *D* and Θ (Figure 9(a)) up to the critical coverage (maximum in Δ*D*) are used to produce a calibration curve relating changes in Θ to changes in *D*: ΔΘ_calibration_(*D_t_*_ <__peak_), as shown in Figure 9(b). Under the reasonable assumption that a given Δ*D* value reflects a unique state of adsorbed vesicles, the fraction of ΔΘ originating from adsorbed vesicles *after* the critical coverage/maximum in *D* was then given by ΔΘ_vesicle_(*t *> peak)= ΔΘ_calibration_(*D_t_*_ > __peak_), as shown in Figure 9(c). By then simply subtracting ΔΘ_vesicle_(*t*) in Figure 9(c) from ΔΘ_total_(*t*), the response of the lipids in the SLB, ΔΘ_SPB_(*t*), is obtained, as shown together with ΔΘ_vesicles_(*t*) in Figure 9(d). With the SPR-signals of liposomes and SLB islands separated, it is possible to apply for example Equation (5) with the appropriate choices of *d* and d*c*/d*n* to each signal respectively. While d*c*/d*n* can often be found in the literature, there is still some confusion about the actual value of d*c*/d*n* for liposomes and SLB, since it is difficult to determine directly (see also Section 7.5 and [93,95] for the influence of anisotropy on the interpretation of d*c*/d*n*). It can also be calculated from theory (see e.g., Cuypers *et al*. [96]) or approximated from waveguide spectroscopy measurements where the optical anisotropy has been taken into account [93]. The effective film thickness can be obtained by visco-elastic modeling of the thickness at the peak in Δ*D* through an iterative approach [14,52,61] or by independent measurements using, e.g., AFM [15]. The result of the mass calculations is shown in Figure 9(e) (see Reimhult *et al*. [14,15] for further details on the calculations) and the sum of the total mass lipid mass—both liposomes and SLB—in Figure 9(f).

One particularly interesting feature that appears in Figure 9(e), is the difference between *m*_vesicle_/*m*_SPB_ and ΔΘ_vesicle_/ΔΘ_SPB_ in Figure 9(d). It is clear from this comparison that a direct transformation of ΔΘ_vesicle_ onto *m*_vesicle_ without taking into account the difference in layer thickness between liposomes and SLB and using a common d*c*/d*n* for a planar bilayer in Equation (5) would result in an underestimation of the vesicle mass by as much as 30%. The dominating contribution to this failure is the difference in d*c*/d*n* between vesicles and planar bilayers, which results from the difference in molecular alignment, but the fact that the thickness is a substantial fraction of the SPR sensing depth must also be considered. Furthermore, by summing up *m*_vesicle_ and *m*_SPB_ (Figure 9(e)), the total lipid mass adsorption, *m*_total_, is obtained as a function of time. In Figure 9(f) *m*_total_ is shown together with the expected mass adsorption from diffusion limited transport of lipid material in liposomes, which now shows a very good agreement with the experimental result.

It is thus clear that, in this particular case, the commonly used method to estimate mass uptake using SPR leads to serious qualitative and quantitative errors. In the present case this was possible to correct for by combining SPR data with the information obtained by combined *f* and *D* measurements using QCM-D. In this way, the possibility to improve the interpretation of evanescent optical field techniques to determine mass uptake is emphasized and is obvious from the detailed kinetics obtained for the two interconnected processes. Perhaps as important is that the combination of QCM-D and SPR, in addition to achieving more quantitative results, resulted in a possibility to separate the response of populations of liposomes and SLB on the sensor surface despite being mean-field methods. This is a qualitatively completely new result and the data in Figure 9(e) can be used to calculate for example the time evolution of the surface coverage of each species [15]. In [15] it is moreover shown how the SPR and QCM-D data can be combined with scanning probe microscopy to further interpret and enhance the modeling of the real-time adsorption process.

In the case of the liposome-to-SLB transformation the separation of species response and the subsequent correction of the quantitative calculations were easily accomplished due to the large contrast between liposomes and SLB islands in the *D*-signal. Even though this is not a general feature of systems undergoing phase or structural transformations, similar procedures will be possible to develop whenever one observable correlates strongly with one conformational state. This occurrence is not too unusual using QCM-D as one technique as the dissipation for highly hydrated states is generally much higher than for collapsed, dehydrated states.

### 7.3. Case Study 3: Real-Time Modeling of Biomolecular Film Properties—A Case Study on SLB Self-Assembly and Protein Adsorption

If data is obtained by two complementary techniques simultaneously in real time or under identical conditions, the kind of analysis presented in Section 7.1 for the PLL-g-PMOXA films can be performed over the entire course of the adsorption process. This can yield insight into how water content and film parameters like viscosity, *μ*, and elasticity, *η*, vary over time during adsorption and conformational transitions. If conformational changes occur which also affect the calculation of the optically determined mass, as for the SLB example above, modeling of the film properties can be done by an iterative process, where the density vector, *ρ_film_*, is used as an input, removing the last unknown parameter in Equation (9), to obtain an effective thickness of the film, *d_film_* [14]. The density has to be a vector, because a calculation is now performed for each correlated time point in the measurement and changes over time since the adsorbate density in the film changes during the process. For SPR measurements, in contrast to in some waveguide measurements, neither the film thickness nor the mass, used in Equation (8), are directly determined by the optical measurement. The new effective thickness can in turn be used as a good approximation for the layer thickness needed to calculate the SPR mass, the dry mass *m_SPR_* in Equation (8) denoted by *m_OWLS._* The entire process is iterated using Equations (8) and (9) until convergence is reached and no further changes in *d_film_* occur [14,46,55]. It has been shown that very fast convergence is typically obtained [14].

**Figure 10:**
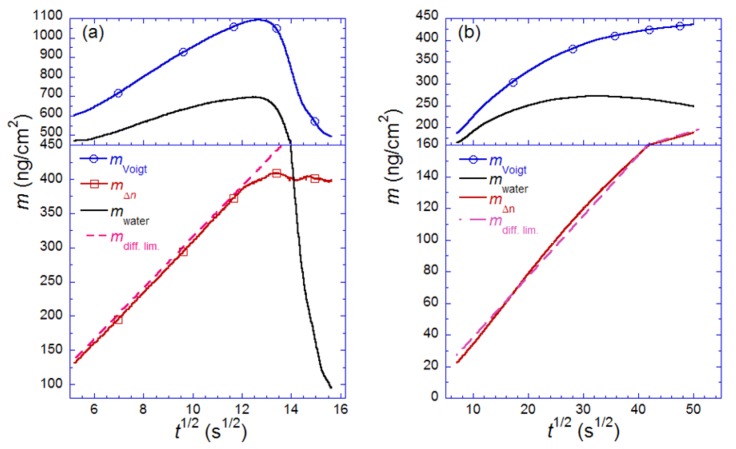
The mass measured by QCM (*m*_Voigt_) and SPR (*m*_Δ*n*_) *vs.* 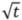 for (**a**) vesicle to bilayer formation; (**b**) streptavidin binding and 2D-crystallization on top of a biotinylated lipid bilayer. Shown is also the difference between the two measured masses, attributed to dynamically coupled water (*m*_water_). The masses are calculated with the iterative method described in the text. Also shown in both plots are the expected adsorption rates for mass-transport limited adsorption (*m*_diff. lim._). Adapted with permission from Reimhult *et al*. [14]. *Anal. Chem*. **2004**, *76*, 7211–7220. Copyright 2004 American Chemical Society.

Figure 10 shows two examples of where this approach has been used: the SLB formation process and for streptavidin binding and possible crystallization on top of the biotinylated SLB [14]. The two processes are chosen for this demonstration, because the SLB formation represents a system undergoing a large structural transformation, while streptavidin is typical for a “simple” protein adsorption process, which is the type of process most often studied.

From an inspection of the liposome-to-SLB transformation (Figure 10(a)), it is clear that the absolute amount of coupled water changes, as expected, most dramatically during the vesicle rupture process (Figure 10(a)) [14]. By comparing *m*_Δ*n*_ to *m*_water_ for the final SLB it is clear that ~20 water molecules are associated with each lipid molecule, corresponding to a water to lipid mass ratio of *φ* ~ 0.5 shown in Figure 10(a). This is a factor of ~1.5 larger than literature values on the hydration of the lipid head groups, which has been estimated to be 11–16 water molecules per phosphocholine lipid [77,78]. However, taking into account that a supported bilayer has been shown to impose local ordering of a layer several water molecules thick [79,80], to be rough in nature and to associate to the surface via a layer of 3–6 water molecules [81], it is clear that the number makes good sense.

Inspection of Figure 10(b) shows that this biologically reasonable ratio only applies at saturation coverage. At low surface coverage *φ* is approximately equal to five. Taking into account that the QCM measures all mass that is coupled to the shear oscillation of the sensor crystal, it is interesting to note that the maximum amount of water that can be entrapped within a ~50 nm in diameter spherical vesicle corresponds to *φ * ≈ 2. In reality, the deformation of the adsorbed vesicles makes the maximum value of *φ* for water trapped in the interior of the vesicle less than one. Since this value is significantly lower than what is observed at low coverage, water in between adsorbed vesicles must also be coupled to the shear oscillation of the sensor crystal.

The same type of decrease in *φ* as function of coverage is observed upon inspection of the streptavidin data (Figure 10(b)). In this case, we are dealing with a protein whose structure is not expected to undergo extensive structural changes during binding, and yet the variation in the amount of coupled water per protein molecule measured by the acoustic QCM sensor varies from *φ* ≈ 7.5 at low coverage to *φ* ≈ 1.3 at high coverage. Assuming that full coverage is reached, *i.e*., close to 50% as has been observed for streptavidin on supported lipid bilayers [46,82], the latter value is in good agreement with complete trapping of essentially all water in between the proteins. Thus, although these measurements show that a large amount of water is part of the QCM-D sensor response also for rigid biomolecular films at full surface coverage—and that water thus comprises a large part of these films—the most striking observation is how dramatically the water content changes and that at low coverage, essentially mostly water is measured by the acoustic sensor [14]. 

Furthermore it has been demonstrated by such analysis that for highly hydrated structures like the liposomes that the viscosity calculated in this way strongly correlates with the viscosity of the layer, while for the significantly denser and thinner streptavidin layer the viscosity instead correlates with the amount of adsorbed protein [14]. A physical basis for this observation was recently proposed from a combined ellipsometry and QCM-D study of adsorbed protein clusters and from a theoretical treatment of hydrodynamic shear flows in films with soft linkers, based on expected differences in water coupling [45,97].

### 7.4. Case Study 4: The Dangers of Jumping to Conclusions Using Single Technique Kinetic Measurements—A Case Study on Bacterial Membrane Mimics

**Figure 11:**
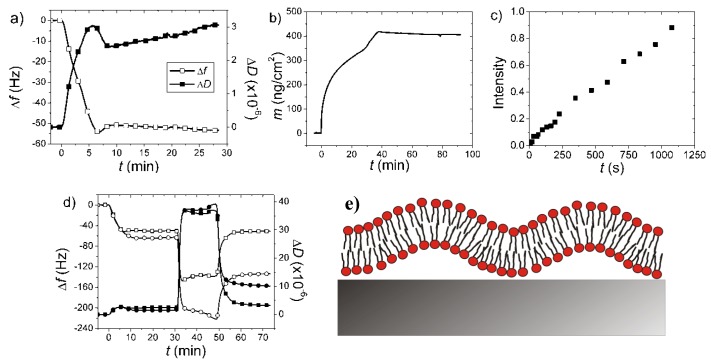
Complementary measurements for *E. coli* total lipid extract liposomes adsorbing to TiO_2_ in the presence of 1 mM CaCl_2_. (**a**) QCM-D frequency (wet mass) and dissipation; (**b**) OWLS (dry) mass; (**c**) Fluorescence recovery after photobleaching of a spot in the adsorbed film; (**d**) Comparison of no adsorption of serum after addition to the adsorbed film on TiO_2_ (Δ*f*, open squares; Δ*D* filled squares) compared to significant adsorption for the partial SLB formed on SiO_2_ (Δ*f*, open circles; Δ*D* filled circles) under the same conditions; (**e**) Probable conformation of the adsorbed lipid film by comparison of the complementary measurements: a non-planar SLB. Reused with permission of Merz, C.; Knoll, W.; Textor, M.; Reimhult, E. [98], adapted from Figure 3 in “Formation of supported bacterial lipid membrane mimics”, *Biointerphases* **2008**, *3*, FA41–FA50. Copyright 2008 AVS. The Science & Technology Society, and with kind permission of Springer Science+Business Media B.V.

Figure 11(a) shows the QCM-D kinetics for adsorption of *E. coli* total lipid extract liposomes to TiO_2_ in the presence of 1 mM CaCl_2_ [98]. Under these conditions a comparison of the kinetics with the established kinetics for SLB formation shown in Figure 8, seems to indicate that here we are observing a process that starts as SLB formation, but then diverges and displays a continuous *increase* in mass and dissipation of the adsorbed layer. This increase seems to continue even after excess liposomes have been removed from the bulk solution. The result seems to indicate an ever more extended film coupled to the sensor substrate. 

In comparison, the OWLS data for the same process in Figure 11(b) also displays the mass transport limited adsorption of lipids with a characteristic kink observed for SLB formation as also observed for SPR raw data, e.g., in Figure 8 [14,15,99]. However, after a slightly higher mass has been adsorbed than expected for the SLB, the OWLS shows a continuously *decreasing* mass in stark contradiction to the QCM-D result. Since these results were highly reproducible and additional measurements could show that the film was laterally fluid (fluorescence recovery after photobleaching shown in Figure 11(c)) and serum resistant (Figure 11(d)), it was concluded that an *E. coli* SLB had been formed under these conditions, but with a non-planar conformation [98]. As described earlier a structure extending from the surface, e.g., an increasingly undulating SLB increasing the mean distance of lipids from the sensor surface and increasing its buffer entrapment, will decrease the OWLS response while it will increase the QCM-D mass loading and dissipation. Thus, using a single biosensor technique to determine the adsorption kinetics for complex processes can be directly misleading, while using a combination with awareness of the different transducer principles, can lead to considering alternative scenarios.

### 7.5. Case Study 5: Analysis of Molecular Ordering and the Influence of Optically Anisotropic Films—A Case Study on Birefringence Analysis of SLBs

In the final example we will show how waveguide spectroscopy can also be used to probe conformational changes in thin biomolecular films by exploiting the inherent anisotropy of films undergoing transitions that affect the molecular order. This was possible by verifying the origin of observed “anomalies” in the acquired data by comparing to QCM-D data obtained for the same adsorption process and adding information from yet one more complementary measurement technique.

If a waveguide has a sensitive enough readout the use of two polarizations for the determination of both refractive index and thickness of the adsorbed film in real time is possible, as was described above. State-of-the-art setups like the Analight Dual Polarization Interferometer (DPI) [100,101,102,103] make such analysis possible. However, in order to determine the two quantities from the measured observables the adsorbed film has to be *isotropic*. This is a condition, which is often not fulfilled for biomolecular films and indeed the opposite is generally true for self-assembly processes, for which molecules with highly anisotropic polarizability are assembled into ordered structures showing preferential alignment with respect to the normal surface. A typical example is again supported planar lipid bilayers which have a molecular arrangement along the normal surface, while their precursor state, liposomes, has an on the average isotropic alignment of the lipid molecules. Thus, for an adsorbed film of liposomes the dominating contribution to the optical response comes from polarization of ionic charges across the entire liposome, while for the SLB a large contribution is due to the difference in molecular polarizability normal to the surface plane and in the surface plane. The transverse magnetic (TM) and transverse electric (TE) linearly polarized optical waveguide modes used to probe the adsorbed film by OWLS or DPI sample the optical properties in different geometrical directions. TM probes the adsorbed film orthogonal to the surface plane and TE additionally in the plane of the film. This leaves three unknown parameters to determine for an SLB: thickness, refractive index and optical anisotropy (or birefringence; the difference between the two main axes of effective refractive indices), but only two observables are typically measured in waveguide spectroscopy (and only one in SPR). A failure to take this into account by complementary measurements will result in the wrong qualitative and quantitative determination of thickness and refractive index and therefore also their product: the adsorbed mass.

**Figure 12:**
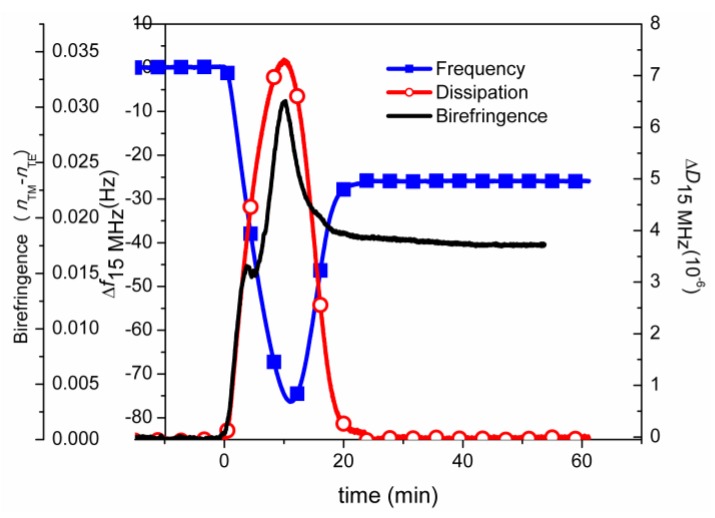
The birefringence evolution for formation of a POPC:POPS (8:2 mol-%) SLB on a SiO_x_N_y_ waveguide in the presence of 2 mM Ca^2+^ compared to the well-known similar kinetics obtained by complementary frequency and dissipation QCM-D measurements. Adapted with permission from Mashaghi *et al*. [95]. *Anal. Chem*. **2008**, *80*, 3666–3676. Copyright 2008 American Chemical Society.

However, if one of the three parameters is estimated using a complementary technique, one can extract even more information about, for example, conformational changes. A recent example is the use of DPI, shown in Figure 12, to calculate the apparent birefringence of a lipid film during liposome adsorption and SLB formation. In this example, we used the fixed thickness of a fully formed SLB as determined by neutron scattering [104] to calculate the refractive index and the birefringence of the adsorbed layer in real time, as the solution as to when both waveguide modes produce the same unique solution to the adsorbed mass and thickness [95]. This procedure, in addition to correcting the mass adsorption kinetics, also makes it possible to follow the conformational changes in the film, which, as described in previous sections, is not clearly evident from normal waveguide spectroscopy and SPR measurements. The black line in Figure 12 shows the birefringence evolution plotted together with the already described QCM-D kinetics for the same process under identical conditions. As can be observed, the birefringence reaches a maximum simultaneously with the dissipation and then decreases to a stable value for the SLB in concert with the QCM-D frequency and dissipation. This shows that extracting the right observable from an evanescent optical sensor using complementary information can increase the sensitivity to conformational changes, since the same behavior is not clearly shown from the mass adsorption kinetics (where it should be absent) or from the thickness kinetics (which is distorted if the anisotropy is not taken into account). 

While in this example the complementary thickness value used to calculate the unknown birefringence (and optical anisotropy) was obtained from neutron scattering experiments, the same procedure could have been carried out using a thickness value obtained through, for example, viscoelastic modeling of the QCM-D response, although this value would not correspond as well with the optical thickness as the one from neutron scattering due to the sensitivity to different physical extensions of the film between acoustic, optical and scattering techniques. Using the kind of complementary measurements just described it is possible to calibrate future measurements of a system using only SPR or waveguide spectroscopy, as was done to obtain the d*n*/d*c* values necessary to do a first interpretation of the SPR response described in Section 7.2 [14]. Table 1 shows calculations of the isotropically averaged refractive indexes of self-assembled SLBs of different compositions obtained by first calculating the birefringence of the films using complementary information on the SLB thickness [95]. The birefringence which is also given in Table 1 is most important in order to avoid misinterpreting the adsorption kinetics in other evanescent optical measurements. As already described in Section 7.2 and Section 7.3, it is especially important when, as in SPR, only linearly p-polarized light is used for sensing and thus sensitivity is only obtained to the refractive index along one of the optical axes in the system. As mentioned earlier, knowing the birefringence also allows an estimate of order in the system [93]. On the other hand, if the birefringence is already known, the refractive index and thickness (total mass) of the system can then be accurately determined [95]. A comparison of e.g., acoustic thickness and optical thickness in real time could now be attempted, but remains to be demonstrated thoroughly.

**Table 1:** Isotropic refractive index, birefringence and thickness for different supported lipid bilayers (SLBs) with standard error of the mean (SEM) given in parenthesis. Thickness in this case refers to the thickness obtained by averaging over the thicknesses obtained by calculating the thickness for a fixed refractive index of the lipids corresponding to 1.47.

	POPC SLB	POPC SLB (Ca^2+^)	POPC:POPS SLB (Ca^2+^)	DOPC SLB	DOPC SLB (Ca^2+^)	DOPC:DOPS SLB (Ca^2+^)
***n***	1.4788 (2.8e–3)	1.4782 (1.2e–3)	1.4711 (1.4e–3)	1.456 (2.5e–3)	1.4693 (4.2e–3)	1.4904 (3.6e–3)
**Birefringence**	0.02164 (5.3e–4)	0.01960 (3.5e–4)	0.01955 (7.1e–4)	0.01586 (7.4e–4)	0.0139 (2.1e–3)	0.0250 (1.9e–3)
**Thickness (nm)**	**4.976 (9.9e** **–2)**	**4.962 (4.3e** **–2)**	**4.689 (5.5e** **–2)**	**3.992 (8.4e** **–2)**	**4.46 (1.4e** **–1)**	**5.2855 (5.1e** **–3)**

## 8. Implications of Case Studies for Sensitivity and Single-Technique Kinetics Measurements

From a measurement perspective a few observations can be made from the time-resolved mass measurements discussed above. First, the relative signal per molecule will differ between an acoustic and an optical sensing technique depending on the water content. The more water within a biomolecular film, the easier it will be to detect adsorbed molecules with an acoustic technique, as this does not have any influence on the signal strength of an evanescent optical technique. On the contrary, the rapidly decaying sensitivity of the optical field perpendicular to the surface reduces its sensitivity to extended, highly water containing, supramolecular complexes. While the sensing depth of e.g., a 4.95 MHz acoustic sensor like the QCM-D is only slightly longer (approximately a factor of two in water), it is increased when a viscoelastic film is adsorbed, while the sensing depth of an optical technique is marginally decreased by adsorption of a higher refractive index film.

Second, a typical feature of acoustic sensing—as demonstrated for the QCM-D—is also the sensitivity to the water content. The dynamically coupled water content is very high at low surface coverage (as demonstrated in Figure 10), which effectively increases the signal strength at low adsorbed surface coverage of the QCM-D. A comparison of how the sensitivity—estimated by the signal-to-noise—differs for some of the different films that have been discussed with different water contents is shown in Table 2. These sensitivities should not be regarded as state-of-the-art sensitivities for the two experimental techniques, rather they are meant to illustrate the trends for sensitivities for different biomolecular films for evanescent optical and acoustic sensors. From Table 2 it is evident how a comparable sensitivity for the two tested optical and acoustic sensors for an SLB with relatively low water content is changed to a relatively much higher sensitivity of the QCM for DNA-films, which almost entirely consist of water. For unsaturated films the difference in sensitivity would be even higher, since *φ* is higher at low coverage. 

**Table 2:** The signal-to-noise ratio for different biomolecular films with varying water content at saturated adsorption as measured using our SPR and QCM-D setups. The relative sensitivity of the acoustic technique increases with water content.

Biofilm	SPR	QCM
***Liposomes***	1,800	4,200
***SLB***	500	630
***Streptavidin***	240	590
***Single 30-mer DNA-strand***	42	380
***fc-hybridization of 30-mer DNA-strand***	**22**	**330**

Third, since the water content changes with molecular surface coverage this also makes it impossible to determine the accurate kinetics, *i.e*., rate of biomolecule adsorption, by using only QCM‑D without additional information. While, for example, the streptavidin data in Figure 11 shows perfect agreement with mass transport limited adsorption for *m*_Δ*n*_, the QCM mass uptake does not follow this adsorption profile. This puts strong doubts on the numerous studies in which the QCM technique alone has been used for estimations of adsorption/binding kinetics or, for that reason, when measured changes in *f* have been used to evaluate differences in the adsorbed amount of biomolecules, without taking the water content into account. The reason is simply that a film composed of a few molecules entrapping a high amount of water will give a response very similar to that of a dense film entrapping a low amount of water.

Fourth, while the sensitivity of an optical evanescent technique in many cases can be increased by adsorbing into a 3D-matrix on the sensor surface extending through the entire sensitivity region of the evanescent field, e.g., the dextran matrix commonly employed by Biacore^®^ for its SPR chips [4], such a strategy is not possible for an acoustic sensor. The adsorbed molecules would, in such a case, replace already trapped water, yielding a low mass load contrast. On the other hand, measuring adsorption of thick, rigid films is possible with the QCM-D, since the extinction depth of the acoustic wave increases with adsorbed visco-elastic overlayers. In the extreme case of rigid films there is no decay of the acoustic wave at all as long as the total mass is <5% of the crystal mass. Polymer layers of a few μm thick can be measured, which for optical surface sensitive methods is only possible using the layer itself as the waveguide medium [105,106].

Fifth, determining the kind of kinetics, e.g., mass transport limited or kinetically limited, from QCM-data is very hard. As above, this is due to the varying amount of trapped water and its properties. However, accurately processed evanescent optical sensor data can easily tell the difference between homogenous films, since mass-transport limited adsorption has a 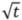-dependence under static conditions and a linear dependence under flow conditions [107], while with one rate-limiting step, kinetically limited adsorption yields a single-exponential time dependence [108]. However, when the type of adsorption is known, this information can be used as an Ansatz to understand the details of the adsorption process by looking at the deviation from the expected behavior in the QCM-D data.

Finally, optical evanescent sensors suffer an equal complication when polarizable molecules in a film undergo strong ordering during adsorption. Under these conditions, the sensitivity can be either increased or decreased depending on type of sensor and alignment of the molecules with the sensor field. Regardless, the effect risks distorting the correct quantitative determination of the adsorbed film properties. A careful kinetic analysis of the adsorption kinetics can also be used in the case of optical sensors, where deviations from expected adsorption behavior can be used instead to infer more complex processes like conformational changes [95].

When comparing evanescent optical and acoustic measurements (or other combinations), it is thus very important to pay attention to the respective limitations of the two techniques, to correct for them using the complementary information when possible, and not to attempt a comparison when the sensitivity of one of the techniques is effectively insignificant.

## 9. Summary

The last ~15 years have seen a tremendous increase in the availability and the sensitivity of methods to characterize biointerfaces and films of biomolecules *in situ*. These developments have been driven by the increased importance of such materials and applications in the rapidly evolving new biology and biotechnology, but also specifically by the need to measure not only the presence of molecules but their often complex arrangement as well. As shown by the examples in this review it has increasingly been realized that to correctly understand the architecture of these thin films it is necessary to understand in detail how the sensor that is used interacts with its sample. It was also shown that since each sensor relies on its separate transducer principle to determine a specific physical property of the film—like mass of adsorbed molecules or film thickness—a correct determination might necessitate the use of more than one sensing technique, because the desired physical property is only rarely measured directly. 

The need for multiple sensing techniques might sound like a drawback and a limitation at first, but with the examples given in this article we also hope to have shown that by combining data from different sensing techniques we can in fact start to study new detailed properties related to the molecular architecture of the film, e.g., time-evolution of hydration, chain stretching, evolution of surface coverage of conformational states, birefringence and molecular ordering. 

The sensor techniques that have so far seen the most combinations with the aim of increasing the understanding of the structure of adsorbed films are combinations of surface sensitive optical and acoustic techniques. Although these techniques are inherently well-suited for complementary measurements a main reason for this combination is the dominance of optical biosensing techniques and the relative high state of commercial development of for example SPR, waveguide spectroscopy and QCM-D. We emphasize that the focus of this review, the combination of evanescent optical sensing techniques like SPR and waveguide spectroscopy with QCM-D are not the only possible choices and in many cases might not even be the best. Recent literature combining e.g., ellipsometry with QCM-D [69,70], surface plasmons with different probing depth [109], fluorescence based imaging and sensing techniques [110,111] or electrochemical sensing methods combined with any of the above to measure function [112,113,114] all have important niches to fill. It is likely that the coming years will continue to see the development of a host of new combinations for the purpose of detailed structural mapping especially of biomolecular films and biointerfacial films. The main driving force for further development will be the increasing focus on not only measuring a response, but relating biomolecular function to structure, which in most cases is also strongly dependent on the structure and properties of the environment in which the molecule is assembled. The addition to the mix of techniques of methods that measure properties locally with high lateral resolution as well as methods to measure charge transfer and molecular alignment will be crucial to measure biomolecular function in adsorbed films in a meaningful way. The recent developments presented here in terms of models and combinations of acoustic and evanescent optical sensing is thus only a prelude to a much wider development that is likely to be both wide-spread and to accelerate in the coming years.
